# Development and assessment of the efficacy and safety of human lung-targeting liposomal methylprednisolone crosslinked with nanobody

**DOI:** 10.1080/10717544.2021.1921073

**Published:** 2021-07-05

**Authors:** Dong Weng, Zhao-Fang Yin, Shan-Shan Chen, Xian He, Nan Li, Tao Chen, Hui Qiu, Meng-Meng Zhao, Qin Wu, Nian-Yu Zhou, Li-Qin Lu, Dan-Li Tang, Jia-Cui Song, Hui-Ping Li

**Affiliations:** a Department of Respiratory Medicine, School of Medicine, Shanghai Pulmonary Hospital, Tongji University, Shanghai, China; b Department of Respiratory Medicine, School of Medicine, Shanghai Pulmonary Hospital, Soochow University, Suzhou, China

**Keywords:** Acute exacerbation of idiopathic pulmonary fibrosis (AE-IPF), humanized surfactant protein-A (hSP-A), humanized surfactant protein-A nanobody (SPANb)

## Abstract

Glucocorticoid (GC) hormone has been commonly used to treat systemic inflammation and immune disorders. However, the side effects associated with long-term use of high-dose GC hormone limit its clinical application seriously. GC hormone that can specifically target the lung might decrease the effective dosage and thus reduce GC-associated side effects. In this study, we successfully prepared human lung-targeting liposomal methylprednisolone crosslinked with nanobody (MPS-NSSLs-SPANb). Our findings indicate that MPS-NSSLs-SPANb may reduce the effective therapeutic dosage of MPS, achieve better efficacy, and reduce GC-associated side effects. In addition, MPS-NSSLs-SPANb showed higher efficacy and lower toxicity than conventional MPS.

## Introduction

The pathology of acute exacerbation of idiopathic pulmonary fibrosis (AE-IPF) is characterized by diffuse alveolar damage (DAD) in addition to usual interstitial pneumonitis (UIP). Clinically, AE-IPF presents as IPF accompanied with acute lung injury (Ryerson et al., 2015). AE-IPF often progresses rapidly and is considered as a seriously life-threatening medical condition, causing high mortality. Song et al. have reported that the one-year and three-year survival of patients with AE-IPF is 56% and 18.4%, respectively (Song et al., 2011). Previous reports have suggested that the inflammatory cascade may be a key mechanism underlying AE-IPF development (Agarwal & Jindal, 2008; Sakamoto et al., 2012). As one of the essential therapies for AE-IPF, glucocorticoid (GC) hormone can manage inflammation effectively and thus rapidly improve respiratory function substantially (Kondoh et al., 1993; Maybauer et al., 2006; Park et al., 2007; Raghu et al., 2011; Wilson & Raghu, 2015). In addition, GC hormone has also been used to treat multiple severe pulmonary inflammatory diseases such as avian influenza-induced severe acute respiratory syndrome and chronic pulmonary diseases, such as sarcoidosis, bronchial asthma, and chronic obstructive pulmonary disease. However, the side effects associated with long-term use of high-dose GC hormone limit its clinical application seriously (Curtis et al., 2006; Judd et al., 2014). As the distribution of GC hormone in the body is not specific and selective, high-dose GC is usually required to achieve satisfactory therapeutic efficacy in the diseased organ, which consequently and unavoidably causes side effects. GC hormone that can specifically target the lung could decrease the effective dosage and thus reduce GC-associated side effects.

Recently, targeted therapy to treat cancer has been developed quickly (Moghimi et al., 2001; Iyer et al., 2015). As liposomes are very stable in serum, they support high-efficient encapsulation and controllable drug release, and can be prepared by standard procedures, they are becoming the most ideal vector for drug delivery (Allen & Cullis, 2013). Liposomes that are cross-linked with antibody can achieve highly specific targeted drug delivery via specific antigen-antibody interaction (Paszko & Senge, 2012; Lin et al., 2015). Of multiple types of antibodies used to modify liposomes, nanobodies^®^ (Nbs) have been considered ideal targeting ligands because of their lower molecular weight, more powerful tissue penetration, higher affinity compared with other types of antibodies (Hamers-Casterman et al., 1993; Siontorou, 2013; Desmyter et al., 2015). Alveolar surfactant protein A (SP-A) is expressed the most abundantly on type II alveolar epithelial cells whereas barely found in the extrapulmonary organs, and thus SP-A is believe to be the most ideal lung tissue target (Kuroki et al., 1998). In our previous study, we have successfully developed anti-rat SP-A Nbs (rSPANb) (Wang et al., 2015) and anti-human SP-ANbs (hSPANb) (He et al., 2017) and have found rSPANbs and hSPANbs target the rat lung and human lung tissues highly specifically. The current study aimed to use hSPANbs as the lung targeting molecule, the clinically commonly used GC hormone methylprednisolone (MPS), and nano-sterically stable liposome (NSSLs) as the drug delivery vector to first develop a GC hormone agent (MPS-NSSLs-SPANb) that specifically targets the human lung. We also tested the toxicity and efficacy of the agent in a rat model of bleomycin (BLM)-induced AE-IPF (Chen et al., 2017). Our study provides experimental evidence for possible clinical translation of the agent.

## Materials and methods

### Materials

1,2-Distearoyl-sn-glycero-3-phosphocholine (DSPC) was obtained from Avanti Polar Lipids, Inc. (Alabaster, AL). 1,2-Distearoyl-sn-glycero-3-phosphoethanolamine-PEG_2000_-COOH (DSPE-PEG_2000_-COOH) and 1,2-distearoyl-sn-glycero-3-phosphoethanolamine-N-[methoxy (polyethylene glycol)-2000] (DSPE-PEG2000) were purchased from Nanocs Inc. (New York, NY). 1-Ethyl-3-(3-dimethylaminopropyl) carbodiimide (EDC), *N-*hydroxy sulfosuccinimide (Sulfo-NHS), 2-(N-morpholino) ethane sulfonic acid (MES), and cholesterol were bought from Sigma-Aldrich (St. Louis, MO). Sepharose CL-4B and sephadex G-25 were provided by EKEAR Biologicals Inc. (Shanghai, China). Humanized surfactant protein-A antigen (hSP-A) was synthesized by Shanghai YouLong Biotech Co. Ltd. (Shanghai, China). Humanized surfactant protein-A nanobody (hSPANb) was synthesized by the Lab of Respiratory Disease, Shanghai Pulmonary Hospital (Shanghai, China). N-(7-nitrobenz-2-oxa-1, 3-diazol-4-yl)-1,2-dihexadecanoyl-snglycero-3-phospoethanolamine, triethylammonium salt (NBD) was obtained from Avanti Polar Lipids, Inc. (Alabaster, AL).

### Preparation of MPS-NSSLs

DSPC, cholesterol, DSPE-PEG_2000_, and DSPE-PEG_2000_-COOH (molar ratio: 20:14.5:1.8:0.05) were dissolved in chloroform/methanol (volume ratio: 2:1). The mixture was dried by nitrogen gas and further dried by speed vacuum overnight. The dried mixture was placed in 0.5 mL water-saturated calcium acetate (200 mM), sonicated for 30 minutes in water bath, and then passed through a liposome extruder (Avanti^®^ Mini-Extruder, Alabaster, AL) attached with a 0.1 μm filter (Nuclepore Track-Etch Membrane, Whatman plc, Kent, UK). The liquid was passed through the liposome extruder repeatedly for 13–17 times. The resulting liposome suspension was kept in a dialysis tube (Float-A-Lyzer G2, Spectra Por, Spectrum Laboratories Inc., Los Angeles, CA) and dialyzed in 0.9% saline at 4 °C overnight. After dialysis, the liposome suspension was kept at 4 °C for future use. The active drug loading method was used to encapsulate MPS with the liposomes under gradient pH condition (Zucker et al., 2009). In brief, MPS was dissolved in 0.9% saline and then mixed with the liposome suspension. The MPS-NSSL suspension was incubated in water bath at 70 °C for 40 minutes and then kept at 4 °C for future use.

Encapsulation efficiency (EE) was analyzed. The MPS-NSSL suspension was aliquoted and kept at 4 °C. At week 0, 4, 8, and 12 of the 4 °C incubation, EE of the MPS-NSSL suspension was analyzed. A total of 0.5 mL MPS-NSSL suspension was loaded on Superose G-25 column (10 × 150 mm), which was equilibrated with 0.001 M PBS contain 0.001 M Na-phosphate and 0.15 M NaCl (pH 7.4). Free MPS (non-encapsulated MPS, MPS_free_) were collected by Superose G25 size-exclusion chromatography. Another 0.5 mL MPS-NSSL suspension was added in 5 mL methanol to dissolve liposomes and release the encapsulated MPS, and total MPS (MPS_total_) were collected by Superose G25 chromatography. MPS_free_ and MPS_total_ were quantified by high-performance liquid chromatography (HPLC) under the following conditions: column: Inertsil C18 (150 mm × 4.6 mm, 5 µm diameter); mobile phase: 0.34% potassium dihydrogen phosphate–methanol (volume ratio: 35:65); column temperature: 300 °C; flow rate: 1 mL/min; wavelength for peak detection 245 nm; sample injection volume: 20 µL. EE was calculated as EE=(MPS_total_ – MPS_free_)/MPS_total_.

### MPS-NSSLs-SPANb preparation and characterization

MPS-NSSL complexes can be crosslinked with humanized SPANb via chemical bond (Manjappa et al., 2011). MPS-NSSL storage buffer was first replaced with the elution buffer (0.1 M MES, 0.5 M NaCl, pH 4–5.5) by Superose G-25 chromatography. Subsequently, 150 µL MPS-NSSL suspension (total 3 μmol liposome complexes) was mixed with 60 μL 0.25 mol/L EDC and 60 μL 0.25 mol/L S-NHS solution (in DDH_2_O) to activate MPS-NSSL complexes. The mixture was incubated at room temperature with gentle stir for 15 minutes, and then was neutralized with NaOH to pH 7.2–7.5. Humanized SPANbs were mixed with the activated MPS-NSSL complexes at different ratios and incubated at 4 °C with gentle stir for eight hours. MPS-NSSLs-SPANb complexes and the unbound SPANbs were then separated by Sepharose CL-4B (10 × 150 mm and pre-equilibrated in 0.001 M PBS) chromatography. MPS-NSSSL-SPANb complexes were analyzed by SDS-PAGE to verify the successful crosslink between SPANbs and MPS-NSSL complexes. Image J (Bethesda, MD) was used to determine the densitometry of the protein bands on the gel, and crosslink efficiency was calculated (Schneider et al., 2012).

The particle size and morphology of MPS-NSSLs-SPANb complexes were determined by laser particle analyzer (NanoZS90, Malvern Instruments, Malvern, UK) and transmission electron microscopy (Cryo-TEM, Tecnai G2 F20, FEI, Eindhoven, The Netherlands), respectively. FITC reacts with antibody protein in alkaline solution, the r-amino group of lysine on protein binds with thiocarbamide of fluorescein to form FITC protein conjugate. Then, the fluorescent labeled antibody was prepared. Liposome fluorescent labeling (NBD) does not need to be coupled. NBD is directly encapsulated into liposomes in the process of making fluorescent labeling liposomes. To observe the morphology, humanized FITC-conjugated SPANbs (SPANb-FITC) were crosslinked to MPS-NSSL complexes according to the description above.

### Determination of MPS-NSSLs-SPANb immunoreactivity by ELISA

MPS-NSSLs-SPANb immunoreactivity was determined by indirect enzyme-linked immunosorbent assay (ELISA). Human SPA antigen (1 µg/mL, 100 µL/well) was added into 96-well ELISA plate, and the plate was incubated at 4 °C overnight. After the plate was washed with PBS buffer for three times, the plate was dried and blocked with 10% calf serum in PBS (150 µL/well) at 37 °C for three times. The plate was then washed with PBS for three times and dried. Humanized SPANb-FITC, MPS-NSSLs-SPANb-FITC, MPS-NSSL, and PBS were added in the plate. After washing with PBS, the plates were incubated with the secondary antibody anti-His-HRP for 45 minutes. Then, the plates were washed with PBS. TMB substrate solution was added to develop color and sulfuric acid was added to terminate the reaction. The absorbance at 450 nm (OD450) was determined in a microplate reader (VARIOSKAN FLASH, Thermo, Waltham, MA).

### Analysis of the specific binding of MPS-NSSLs-SPANbs to human lung tissues by immunohistochemistry

Frozen sections of human lung, liver, spleen, and kidney tissues were prepared and stained with humanized SPANb with His tag (positive control), MPS-NSSLs-SPANb with His tag, MPS-NSSLs, and PBS (negative control). Anti-His monoclonal antibody and HRP-conjugated antibody were used for immunohistochemistry.

### Analysis of MPS-NSSLs-SPANb distribution in nude mice by *in vivo* imaging

As the SPA amino sequences of human and nude mice share a very high homology (95%), we used *in vivo* imaging technology to assess humanized SPANb-FITC distribution in nude mice. Five nude mice were anesthetized by isoflurane inhalation and then injected via the tail vein with equal amount of SPANb-FITC, MPS-NSSLs-SPANb-FITC, NSSL-SPANb-FITC, MPS-NSSLs-NBD, and NSSLs-NBD (1 mg/kg), respectively. Fifteen minutes, 1 h, 3 h, 6 h, and 8 h after the injections, the small animal imaging system (NightOWL LB-983, Berthold, Bad Wildbad, Germany) was used to detect real-time fluorescence signal distribution in the nude mice.

### MPS-NSSLs-SPANb distribution in rats

A total of 105 healthy SD male rats were randomized into three groups (35 rats/group): MPS-NSSLs-SPANb, MPS-NSSLs, and MPS groups. The dose of all injections was 2 mg/kg body weight. Fifteen minutes, 30 min, 1 h, 2 h, 4 h, 8 h, and 12 h after the injections, the rats were anesthetized by isoflurane inhalation and then sacrificed (*n* = 5, at each time point). Bloods were collected from the orbital after the eye balls were removed, and the heart, liver, spleen, lung, and kidney were dissected. The blood samples were mixed with EDTA and centrifuged to collect serum. The tissue specimens were washed with 0.9% NaCl saline, dried, accurately weighted, and kept at −20 °C for future use. MPS levels in the serum and tissue specimens were determined by HPLC.

To assess the targeting efficiency of MPS-NSSLs-SPANb, peak concentration ratio (Ce) and comparative uptake (Re) were used to assess the tissue distribution of MPS-NSSLs-SPANb and MPS-NSSLs. Ce=(Cp)a/(Cp)b. Cp represents peak concentration; a represents MPS-NSSLs-SPANb or MPS-NSSLs group; b represents MPS group. Ce represents the differences in drug distribution of the two groups. Higher Ce values correlate with greater differences in tissue distribution of the two groups. Re=(AUCi)a/(AUCi)b. AUCi (area under ROC curve) represents the AUC of the concentration–time ROC curve of organ ‘i’, which was calculated by the OringinPro 9.0 software. Re> 1 represents that the drug can target the organ ‘i’; higher Re is associated with more effective targeting; Re < 1 represents no tissue or organ specific targeting of the drug.

### Therapeutic effect of MPS-NSSLs-SPANb on rats with BLM-induced AE-IPF

A total of 120 male SD rats (weighted 90 ± 5 g) were randomized into six groups: (A) regular-dose MPS-NSSLs-SPANb (MPS 1 mg/kg)+AE-IPF group, (B) low-dose MPS-NSSLs-SPANb (MPS 0.5 mg/kg)+AE-IPF group, (C) MPS-NSSLs (MPS 1 mg/kg)+AE-IPF group, (D) MPS (MPS 1 mg/kg)+AE-IPF group, (E) AE-IPF only group, and (F) normal control group. Each group was further divided into two subgroups: one-week exposure and two-week exposure (10 rats in each subgroup). AE-IPF was established by two intratracheal injection with BLM in rats under a laryngoscope (Chen et al., 2017). The normal control group was injected with saline in the similar manner. EE was determined for each batch of MPS-NSSLs-SPANb preparation as mentioned before. MPS-NSSLs-SPANbs with satisfactory EE were used for animal experiments. Drug-exposure details in each group are described in Table S6.

Rats were sacrificed after one-week or two-week exposure to the drugs. The middle lobe of right lung was dissected, fixed in 10% formalin for 48 hours, embedded in paraffin, and sectioned. The tissue sections were used for hematoxylin–eosin (H&E) staining and Masson staining. The staining images were analyzed under the Leica SCN400 (400× magnification, Leica Biosystems, SCN400, Nußloch, Germany). According to the Mikawa K method, H&E staining was scored (Mikawa et al., 2003). Three observational fields were randomly selected from each Masson-staining image, and the percentage of staining area was analyzed by the image analysis software Image-Pro Plus 6.0 (Bethesda, MD).

Inflammatory factor levels in bronchoalveolar lavage fluid (BALF) were determined by ABC-ELISA. The left lung of each rat was perfused (1 mL × 4). The BALF was then collected and centrifuged at 3000 rpm at 4 °C for five minutes. The supernatants were collected and analyzed by ABC-ELISA.

NF-κB mRNA expression in lung tissues was determined by RT-PCR. Total RNA of the anterior lobe of right lung was extracted. Real-time PCR was performed to determine NF-κB mRNA levels. The relative NF-κB mRNA levels were calculated according to the equation: 2^–Δct^×100%, Δct = cycle threshold (CT) of the target gene – CT of the internal reference (β-actin). Primer sequences are listed in Table S7.

Lung water content was measured to determine pulmonary edema. The posterior lobe (250 mg) of right lung of each rat was collected and freeze dried in a freeze-dryer (Beijing Sihuan Company, LGJ-10D, Beijing, China) overnight. The weight differences between wet and dried lung tissues represent lung water contents. Lung water content = wet weight – dry weight.

To estimate the safety of MPS-NSSLs-SPANb, 120 SD male rats were randomized into the six groups as the description above. Rats were sacrificed after one-week or two-week exposure. Bloods were collected from abdominal vein and the serum was separated. To estimate liver and kidney function, serum levels of alanine aminotransferase (ALT), aminotransferase (AST), urea nitrogen (BUN), and creatinine (CR) were measured in an automated biochemical analyzer (HITACHI, Automatic Analyzer 7600-110, Tokyo, Japan). After the rats were sacrificed, the lung was perfused under sterile condition. The perfusion buffer was collected and cultured to estimate bacterial and fungal infection.

A total of 90 male SD rats (weighted 90 ± 5 g) were randomized into the five AE-IPF groups as the description above and treated as the description in Table S6. Rat survival was observed after one-week and two-week exposure.

### Statistical analysis

The statistical analysis software GraphPad prism 5 (La Jolla, CA) was used for all of the statistical analyses. Continuous variables are presented as mean ± standard deviation (SD). Multiple group comparison was analyzed by ANOVA. Inter-group comparisons were performed using the independent sample *t*-test. Survival curve was plotted using the Kaplan–Meier method, and the survival time was compared by the log-rank test. *p* < .05 was considered significantly different.

## Results

### MPS-NSSLs-SPANb physicochemical characteristics

**Figure 1.**
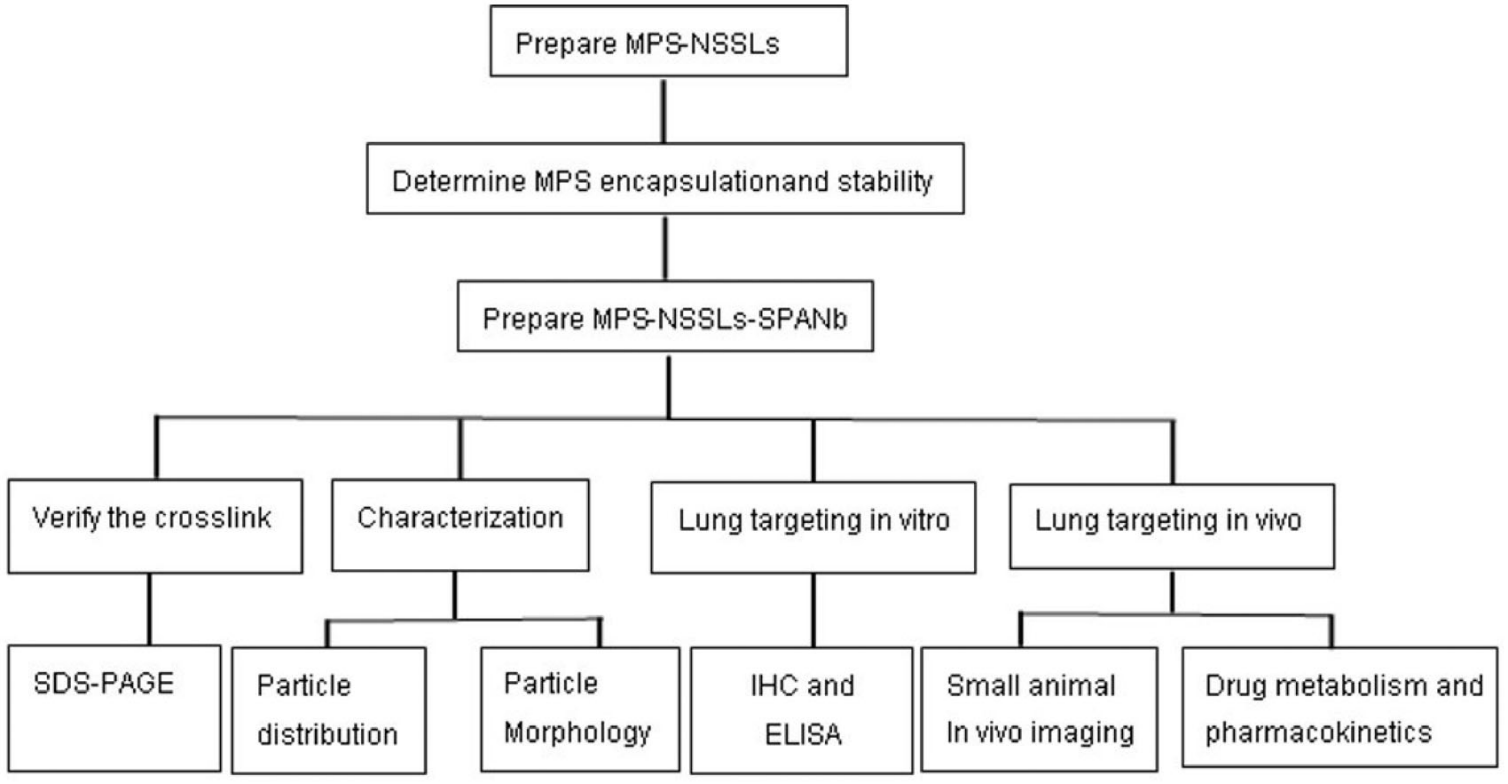
Experimental design.

**Figure 2.**
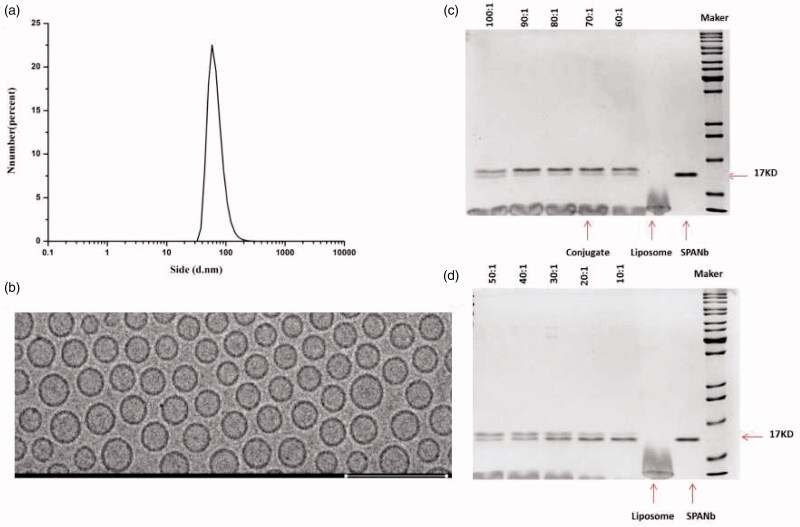
MPS-NSSLs-SPANb physicochemical characteristics and image of SDS-PAGE gel showing SPANb, NSSLs, and MPS-NSSLs-SPANb. (a) Particle size distribution of MPS-NSSLs-SPANb. The mean particle size is 89 ± 0.2 nm. (b) A Cryo-TEM image of MPS-NSSLs-SPANb. The scale bar represents 200 nm. (c, d) Humanized SPANb and liposomes (MPS-NSSLs) were mixed at the indicated molar ratios and crosslinked. The crosslink reaction products were loaded on a SDS-PAGE gel. The arrows are pointing to humanized SPANb, liposomes (MPS-NSSLs), and MPS-NSSLs-SPANb (MPS-NSSLs: humanized SPANb = 70:1), respectively.

The overall experimental design is displayed in Figure 1. EE immediately after the encapsulation reaction was 90.06%±0.32% and was not significantly affected when the reaction products were kept at 4 °C for up to 12 weeks (*p* > .05, Table S1), suggesting that MPS-NSSL complexes appear stable at 4 °C. The mean particle size of MPS-NSSLs-SPANb was 89 ± 0.2 nm, and the size range of MPS-NSSLs-SPANb particles was fairly narrow (Figure 2(a)). Cryo-TEM showed that MPS-NSSLs-SPANb particles were in regular spherical shape and dispersed well (Figure 2(b)). MPS-NSSLs-SPANbs appeared as a protein band with a greater molecular weight than SPANbs on SDS-PAGE gel (Figure 2(c,d)), indicating the successful crosslink of MPS-NSSLs and humanized SPANbs. Crosslink reaction was optimal when the ratio of humanized SPANbs to MPS-NSSLs was 1:70 to 1:90. The crosslink efficiency was 66%±5.2% when the ratio of humanized SPANbs: MPS-NSSLs was 1:70.

### MPS-NSSLs-SPANb targeted the lung

**Figure 3.**
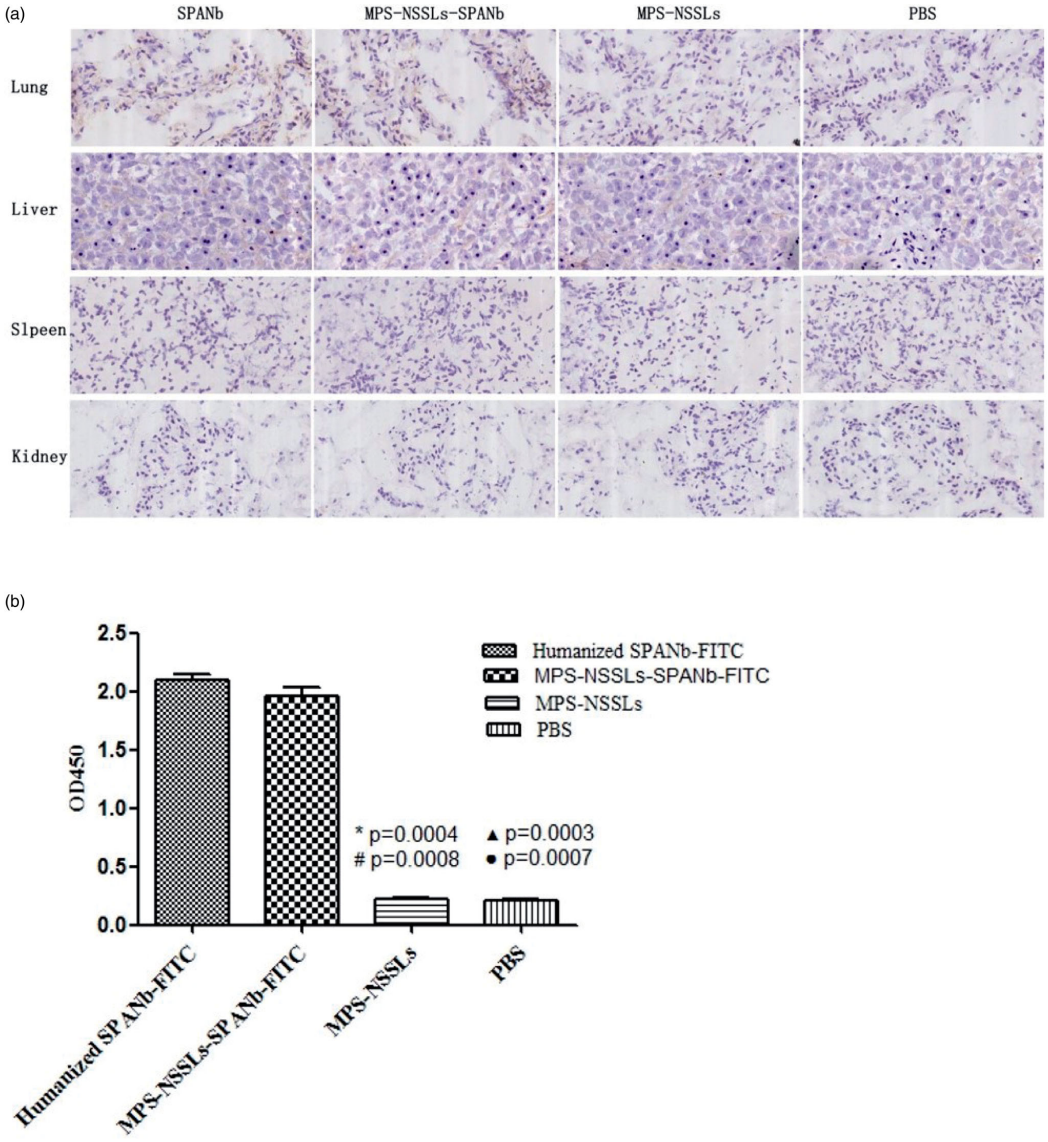
Images of immunohistochemical staining of human tissue specimens and *in vitro* binding to antigen SPA by MPS-NSSLs-SPANb. (a) The magnification was ×20. Arrows are pointing to positive staining. (b) The ELISA plate was coated with recombinant human SPA protein. MPS-NSSLs-SPANb-FITC, humanized SPANb-FITC (positive control), MPS-NSSLs, and PBS (negative control) were added to the plate. *Significantly different between humanized SPANb-FITC vs. MPS-NSSLs. ^▲^Significantly different between humanized SPANb-FITC vs. PBS. ^#^Significantly different between MPS-NSSLs-SPANb-FITC vs. MPS-NSSLs. ^●^Significantly different between MPS-NSSLs-SPANb-FITC vs. PBS.

**Figure 4.**
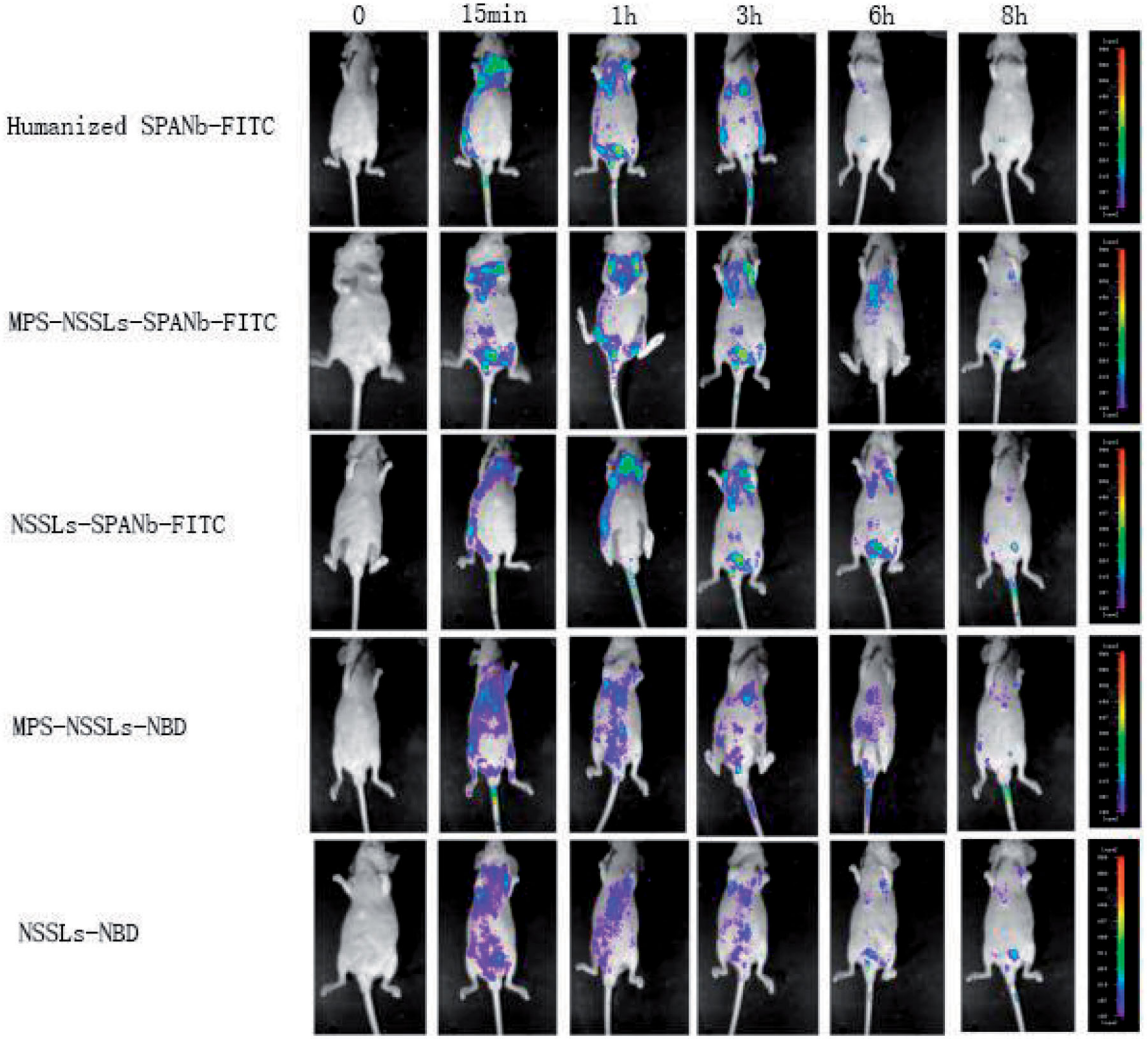
Real-time *in vivo* imaging of nude mice injected with different agents. *In vivo* imaging showed that MPS-NSSLs-SPANb-FITC and humanized SPANb-FITC apparently accumulated in the lung of nude mice 15 minutes after being injected in the nude mice, and the pulmonary accumulation remained substantial 3 h after the injections. The experiment was repeated three times. Red arrows are pointing to the pulmonary accumulation of the agents.

IHC found that MPS-NSSLs-SPANbs and humanized SPANbs bound human lung tissue specifically but did not react with human liver, spleen, and kidney tissues (Figure 3(a)), suggesting that MPS-NSSLs-SPANb may target human lung specifically. MPS-NSSLs and PBS did not bind any of the human tissues (Figure 3(a)). Indirect ELISA showed that MPS-NSSLs-SPANb-FITC bound human SPA antigen as effectively as the positive control humanized SPANb-FITC (*p*> .05), and the binding was significantly higher than that of MPS-NSSLs and the negative control PBS (*p* < .01, Figure 3(b)). *In vivo* imaging showed that MPS-NSSLs-SPANb-FITC and humanized SPANb-FITC apparently accumulated in the lung of nude mice 15 minutes after being injected in the nude mice, and the pulmonary accumulation remained substantial 3 h after the injections (Figure 4). The excretion pattern of MPS-NSSLs-SPANb-FITC and humanized SPANb-FITC was similar in nude mice. MPS-NSSLs-NBD and NSSLs-NBD did not show lung-specific accumulation (Figure 4).

**Figure 5.**
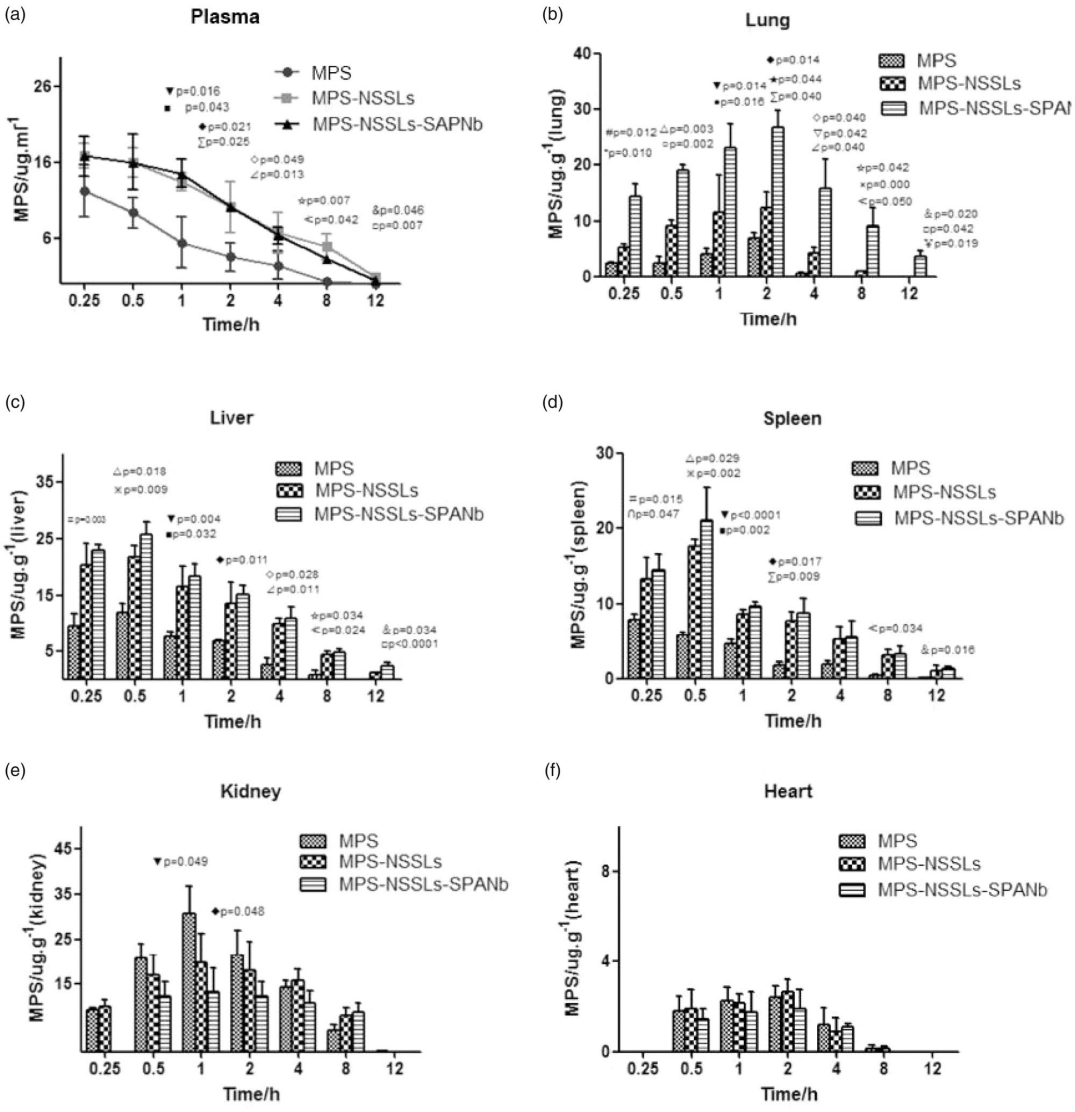
MPS levels in plasma and other tissues of rats injected with MPS, MPS-NSSLs, or MPS-NSSLs-SPANb. MPS-NSSLs-SPANb, MPS-NSSLs, or MPS were injected to rats via the tail vein. Rats were sacrificed at different time points. MPS levels in plasma and rat tissues were determined by HPLC. ^#△▼◆◇☆&^Significantly different between MPS-NSSLs-SPANb vs. MPS at 0.25 h, 0.5 h, 1 h, 2 h, 4 h, 8 h, and 12 h. ^*○●★▽×□^Significantly different between MPS-NSSLs-SPANb vs. MPS-NSSLs at 0.25 h, 0.5 h, 1 h, 2 h, 4 h, 8 h, and 12 h. ^∩※■∑⊿≮￥^Significantly different between MPS-NSSLs vs. MPS at 0.25 h, 0.5 h, 1 h, 2 h, 4 h, 8 h, and 12 h.

To investigate whether MPS-NSSLs-SPANb can target the lung in rats, we measured MPS levels in rat organs. MPS circulation time and plasma MPS levels in the MPS-NSSLs-SPANb and MPS-NSSLs groups were longer and higher than those of the MPS group (Figure 5(a)). Plasma (Figure 5(a)) and pulmonary MPS levels (Figure 5(b)) in the MPS-NSSLs-SPANb group were significantly higher than those in the MPS group at all-time points after injection (*p* < .01). In lung tissues, although MPS levels of the MPS-NSSL group were significantly higher than those of the MPS group at 15 min, 30 min, 1 h, 2 h, 4 h, and 8 h after the injections (*p*<.01), MPS levels in the MPS-NSSLs-SPANb groups were the highest at all-time points (Figure 5(b)). In liver (Figure 5(c)) and spleen (Figure 5(d)) tissues, MPS levels in the MPS-NSSLs-SPANb and MPS-NSSLs groups were similar but significantly higher than those in the MPS group (*p* < .05). In contrast to other tissues, in kidney tissues, MPS levels in the MPS-NSSLs-SPANb group were significantly lower than those in the MPS group at 1 h and 2 h after the injections (*p* < .05, Figure 5(e)). In heart, MPS levels were similar in the MPS, MPS-NSSLs, and MPS-NSSLs-SPANb groups at all-time points (Figure 5(f)). These findings clearly support that MPS-NSSLs-SPANb accumulates in the lung specifically.

**Figure 6.**
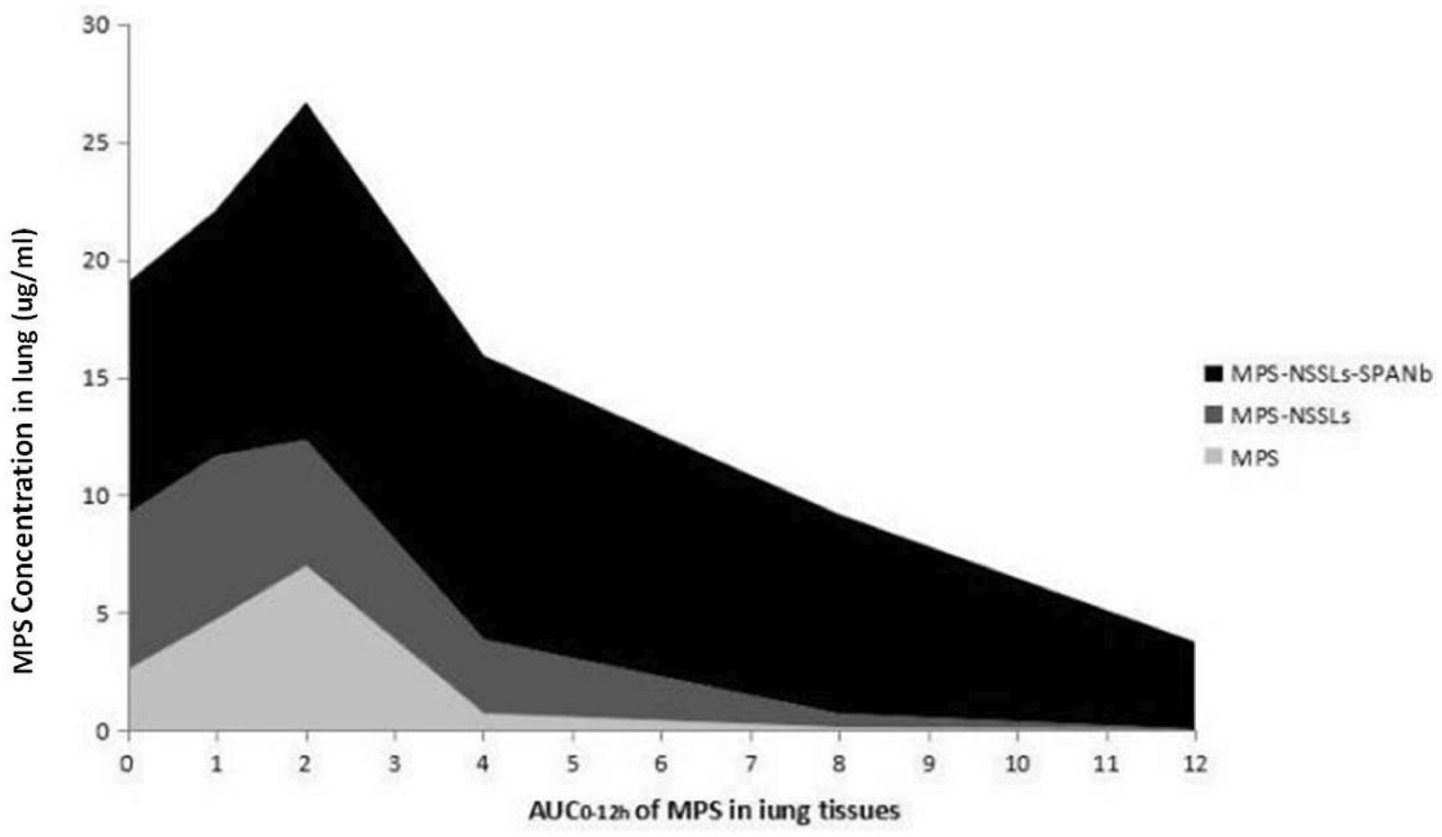
AUC_0–12 h_ of MPS levels in lung tissues. MPS-NSSLs-SPANb, MPS-NSSLs, or MPS were injected into rats via the tail vein. MPS levels in lung tissues were determined by HPLC. AUC_0–12 h_ of MPS was 17.16 μg/g. AUC_0–12 h_ of MPS-NSSLs-SPANb group was 158.19 μg/g. Re = 9.22.

Analyses of AUC further supported that MPS-NSSLs-SPANb targeted lung tissue effectively (Tables S2 and S3, Figure 6). MPS peak concentration in lung tissues in the MPS-NSSLs-SPANb group was 3.81 times of that in the MPS group (Ce = 3.81, Table S3). The AUC_0–12 h_ of the MPS-NSSLs-SPANb group was 9.22 times of that of the MPS group (Re = 9.22, Table S3). MPS-NSSLs and MPS-NSSLs-SPANb were also enriched slightly in the liver and spleen, but did not accumulate in the heart and kidney (Table S3).

### MPS-NSSLs-SPANb caused minimal adverse effects in rats with AE-IPF

After one-week and two-week exposure to the drugs, serum levels of ALT, AST, BUN, and Cr in regular-dose MPS-NSSLs-SPANb (MPS 1 mg/kg) and low-dose MPS-NSSLs-SPANb (MPS 0.5 mg/kg)+AE-IPF groups were similar as those in the normal control group (Table S4). Notably, serum ALT and Cr levels in the MPS + AE-IPF two-week exposure group were significantly higher than those in the normal control group (*p* < .05, Table S4), indicating that MPS may cause liver and kidney toxicity. Compared with the normal control group, the AE-IPF group exhibited significantly higher serum BUN levels one-week after AE-IPF induction and higher serum ALT and Cr levels two-week after AE-IPF induction (*p* < .05, Table S4). The MPS-NSSLs + AE-IPF one-week exposure group had one case of positive *Staphylococcus epidermidis* from BALF culturing (Table S5). The regular-dose MPS-NSSLs-SPANb (MPS 1 mg/kg)+AE-IPF two-week exposure group had one case of positive *S. epidermidis*; the MPS-NSSLs + AE-IPF two-week exposure group had one cases of positive *S. epidermidis*, two cases of positive *E. coli*; the MPS + AE-IPF group had three cases of positive *S. epidermidis* (Table S5). These data indicate MPS-NSSLs-SPANb may not increase rats’ susceptibility to bacterial infection.

### MPS-NSSLs-SPANb attenuated the adverse effects of AE-IPF and extended survival in rats with AE-IPF

**Figure 7.**
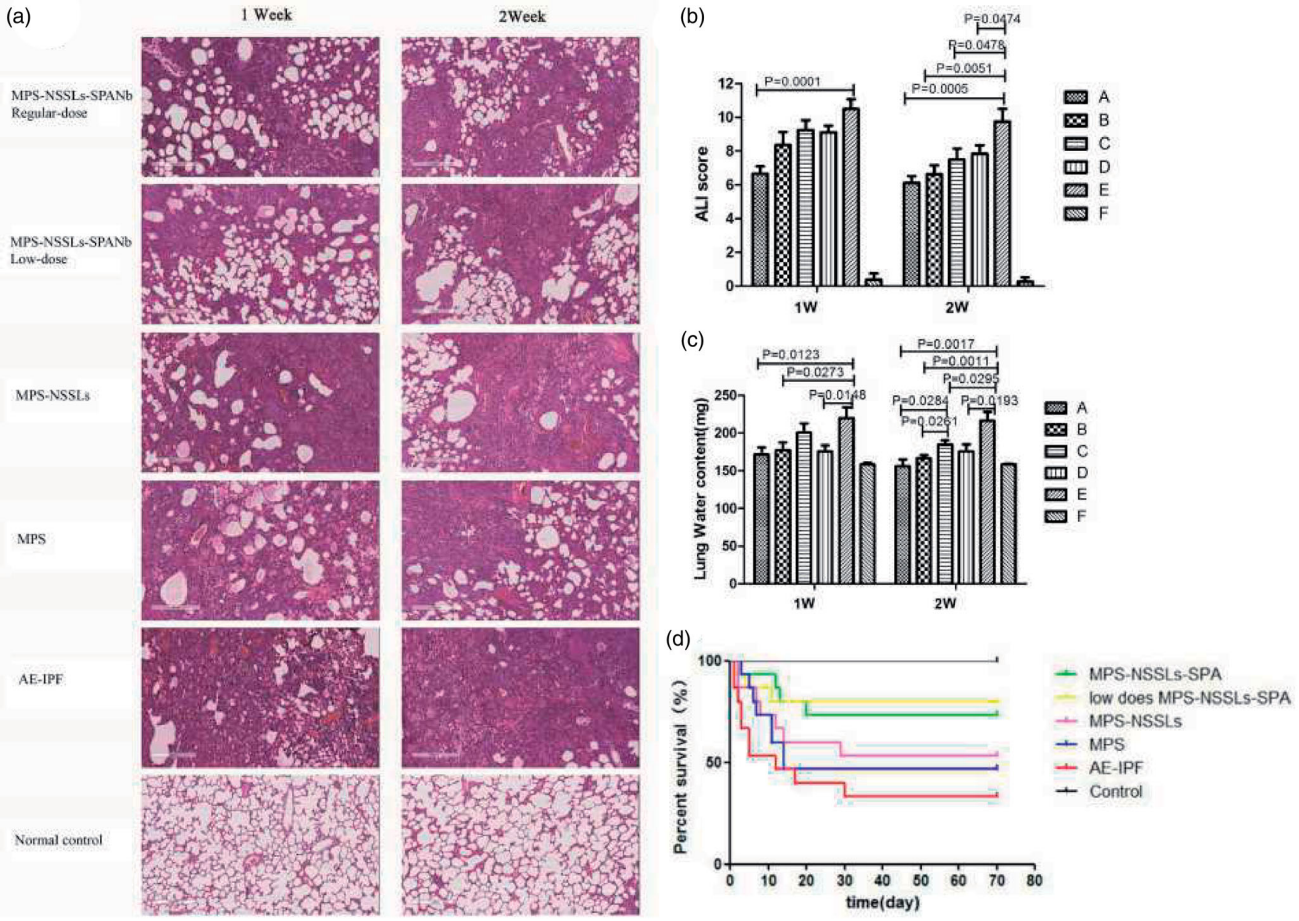
Effects of different agents on lung tissue damage and survival in rats with AE-IPF. (a) Rat tissues were sectioned and stained with H&E. The magnification was ×20. (b) ALI score of rat lung tissues. (c) Lung water contents in rats with AE-IPF. (A) Regular-dose MPS-NSSLs-SPANb (MPS 1 mg/kg)+AE-IPF group; (B) low-dose MPS-NSSLs-SPANb (MPS 0.5mg/kg)+AE-IPF group; (C) MPS-NSSLs + AE-IPF group; (D) MPS + AE-IPF group; (E) AE-IPF group; (F) normal control group. (d) The 10-week survival rate of the regular-dose (MPS 1 mg/kg) and low-dose MPS-NSSLs-SPANb (MPS 0.5 mg/kg)+AE-IPF group was 73.33% and 80.00%, respectively, and was significantly higher than the AE-IPF group (33.33%). *p*=.0218, regular-dose MPS-NSSLs-SPANb (MPS 1 mg/kg)+AE-IPF vs. AE-IPF. *p*=.0135, low-dose MPS-NSSLs-SPANb (MPS 0.5 mg/kg)+AE-IPF vs. AE-IPF.

H&E staining revealed that the AE-IPF group exhibited abnormal alveolar structure, thickened alveolar wall, obvious inflammatory cell infiltration, pulmonary congestion, and transparent membrane formation (Figure 7(a)). The regular-dose (MPS 1 mg/kg) and low-dose MPS-NSSLs-SPANb (MPS 0.5 mg/kg)+AE-IPF one-week exposure groups showed considerably reduced inflammation compared with the AE-IPF group. All the groups exposed two-week to any types of MPS showed significantly attenuated pulmonary tissue damage compared with the AE-IPF group, (*p* < .05, Figure 7(b)). Masson staining showed that the AE-IPF group had excessive pulmonary collagen deposition and all the groups exposed to any types of MPS still exhibited large pulmonary collage deposition (Figures S1 and S2) although inflammation appeared to be reduced compared with the AE-IPF group.

Lung water contents were significantly reduced in the regular-dose (MPS 1 mg/kg), low-dose MPS-NSSLs-SPANb (MPS 0.5 mg/kg), and MPS + AE-IPF one-week exposure groups compared with the AE-IPF group (*p* < .05, Figure 7(c)). After two-week exposure, all of the groups exposed to any types of MPS had significantly lower lung water contents compared with the AE-IPF group (*p* < .05, Figure 7(c)). These data suggest MPS may relieve pulmonary edema in AE-IPF.

Ten-week survival rate of the regular-dose (MPS 1 mg/kg) and low-dose MPS-NSSLs-SPANb (MPS 0.5 mg/kg)+AE-IPF groups was 73.33% and 80.00%, respectively, which were significantly higher than that of the AE-IPF group (33.33%, *p*= .0218 vs. the regular-dose group, *p*= .0135 vs. the low-dose MPS-NSSLs-SPANb + AE-IPF group, Figure 7(d)). Although the MPS-NSSLs + AE-IPF and MPS + AE-IPF groups had higher survival rates than the AE-IPF group, the increases were not significant (Figure 7(d)). These findings indicate that MPS-NSSLs-SPANb may have a better therapeutic efficacy than MPS to treat AE-IPF.

**Figure 8.**
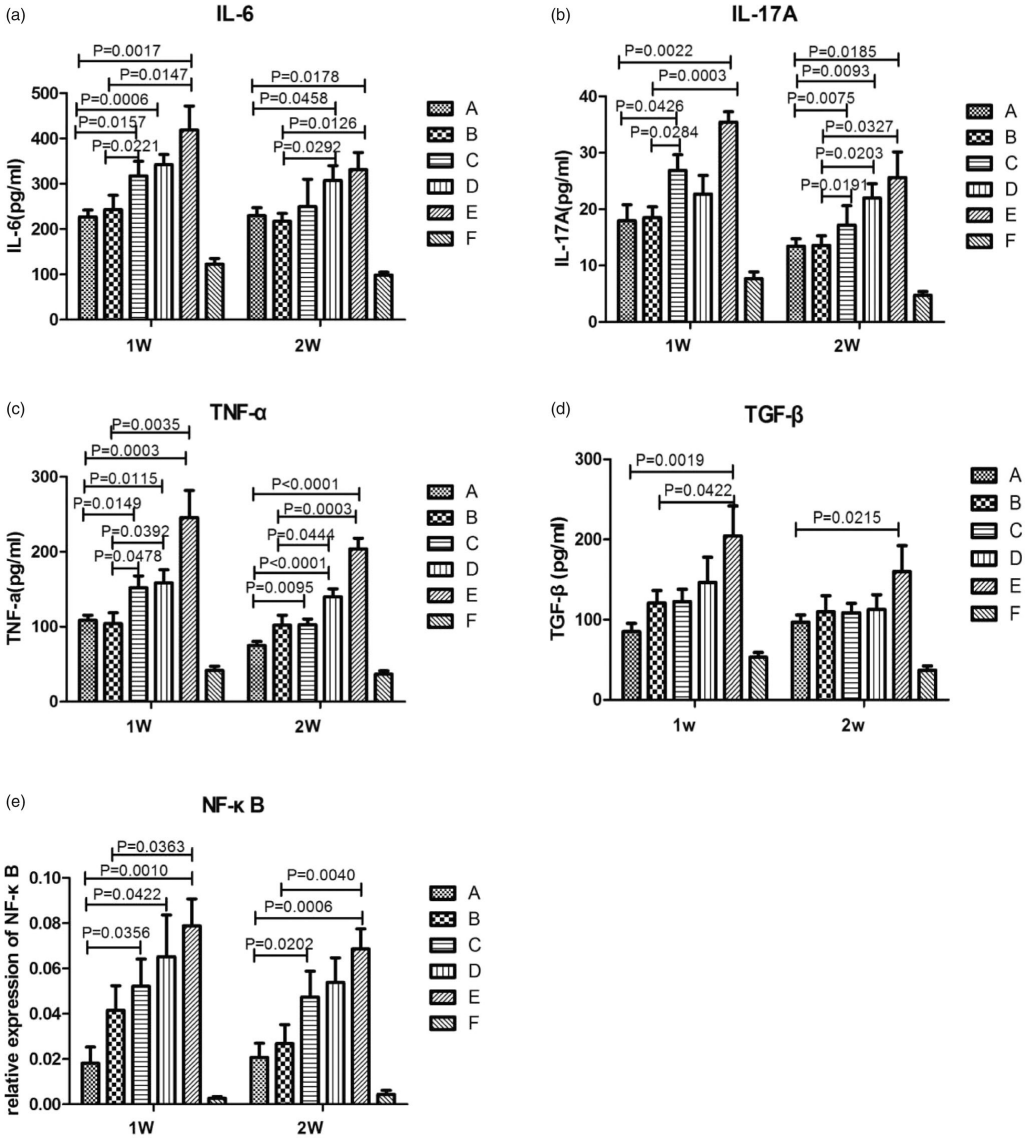
IL-6, IL-17A, TNF-α, and TGF-β levels in bronchoalveolar lavage fluid and NF-κB mRNA expression in lung tissue of rats with AE-IPF. (a) IL-6 levels in BALF. (b) IL-17A levels in BALF. (c) TNF-α levels in BALF. (d) TGF-β levels in BALF. (e) NF-κB mRNA expression in rat lung tissues. (A) Regular-dose MPS-NSSLs-SPANb (MPS 1 mg/kg)+AE-IPF group; (B) low-dose MPS-NSSLs-SPANb (MPS 0.5 mg/kg)+AE-IPF group; (C) MPS-NSSLs + AE-IPF group; (D) MPS + AE-IPF group; (E) AE-IPF group; (F) normal control group.

The levels of IL-6, IL-17A, TNF-α, and TGF-β in BALF were significantly higher in the AE-IPF group than in the normal control group (*p* < .05, Figure 8). All the groups exposed to any types of MPS had significantly reduced IL-6, IL-17A, TNF-α, and TGF-β levels in BALF compared with the AE-IPF group (*p* < .05, Figure 8). In addition, the regular-dose (MPS 1 mg/kg) and low-dose MPS-NSSLs-SPANb (MPS 0.5 mg/kg)+AE-IPF groups showed significantly reduced IL-6, IL-17A, and TNF-α levels compared with the MPS + AE-IPF group (*p* < .05, Figure 8(a–c)), and the regular-dose (MPS 1 mg/kg) and low-dose MPS-NSSLs-SPANb (MPS 0.5 mg/kg)+AE-IPF one-week exposure groups had the lowest levels of the three inflammatory cytokines (Figure 8(a–c)). TGF-β levels were similar in the groups exposed to two-week any-type of MPS (Figure 8(d)). These results indicate that MPS-NSSLs-SPANb appear to have stronger anti-inflammatory effects but may not further reduce pulmonary fibrosis compared with MPS.

Lung NF-κB mRNA expression in the AE-IPF group was significantly higher than that in the normal control group (*p* < .05, Figure 8(e)). The regular-dose MPS-NSSLs-SPANb (MPS 1 mg/kg)+AE-IPF one-week exposure group had significantly reduced pulmonary NF-κB mRNA expression than the MPS-NSSLs + AE-IPF, MPS + AE-IPF, and AE-IPF groups (*p* < .05, Figure 8(e)). After two-week exposure, both the regular-dose (MPS 1 mg/kg) and low-dose MPS-NSSLs-SPANb (MPS 1 mg/kg)+AE-IPF groups exhibited significantly decreased NF-κB mRNA expression than the AE-IPF groups and had the lowest NF-κB mRNA expression (*p* < .05, Figure 8(e)).

## Discussion

**Figure 9.**
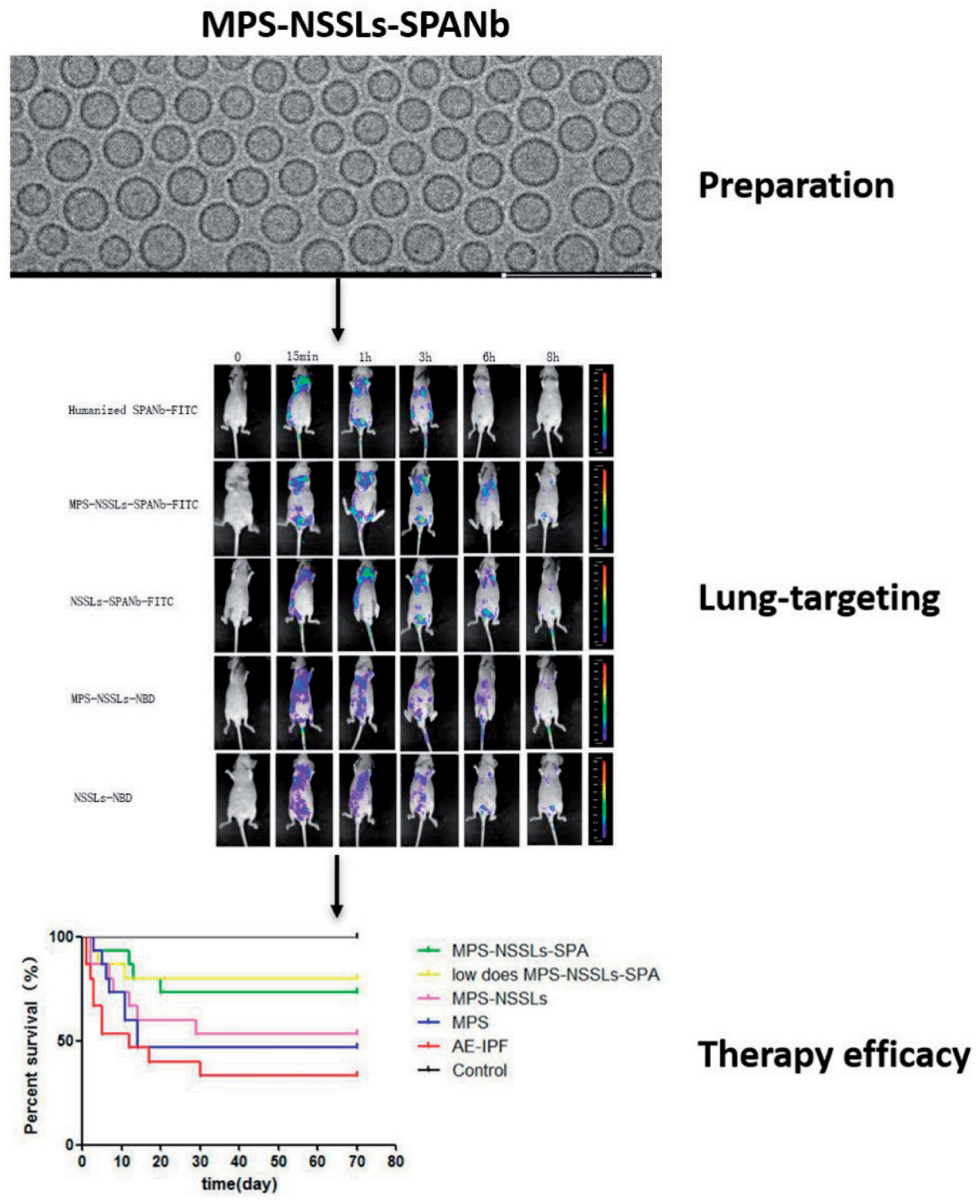
Graphic table of contents.

Compared with conventional liposomal drugs, active targeting liposomal drugs are more specific to the therapeutic targets, support better efficacy, and have lower toxicity (Wicki et al., 2015). Due to its abundant pulmonary expression but minimal expression in other tissues, SP-A is thought to be highly specific to the lung and thus can serve as an ideal target to the lung (Hamers-Casterman et al., 1993). Here, we used our previously developed nanobody to human lung SP-A (SPANb) (He et al., 2017), NSSLs as the drug delivery system, and MPS as the therapeutic drug to successfully prepare the human lung targeting GC drug MPS-NSSLs-SPANb (Figure 9).

MPS-NSSLs-SPANb particles developed in the current study exhibit obvious advantages. For the preparation of the particles, membrane dispersion was combined with sonication and film extrusion methods to prepare PEG liposomes. The resulting MPS-NSSLs-SPANb particles were dispersed perfectly and had an average particle size of 89 ± 0.2 nm, which supports powerful tissue penetration (Koshkaryev et al., 2013). In addition, liposomes embedded in PEG can reduce their uptake by the reticular endothelial system so to extend the circulation time of the liposomes (Torchilin et al., 1994). Furthermore, we used gradient pH to actively encapsulate MPS into the liposomes and achieve maximal MPS encapsulation (EE > 90%). The MPS-NSSL complexes were stable at 4 °C for up to 12 weeks. Humanized SPANb was used as the lung targeting agent and crosslinked to the MPS-NSSLs by amino-carboxyl chemical bonds, which are very stable, reproducible, and evenly distributed on the surface of SPANb and MPS-NSSLs. The crosslink reaction was mild, efficient, and highly specific.

The current study provided several lines of evidence to support that the MPS-NSSLs-SPANbs can target the lung specifically and effectively. First, both MPS-NSSLs-SPANbs and the positive control humanized SPANb bound human lung tissues with a similar effectiveness whereas did not react to human tissues from other organs. Second, MPS-NSSLs-SPANb-FITC accumulated in the lung of nude mice 15 minutes after the agent was injected into the nude mice and remained enriched in the lung three hours after the injection. In contrast to these results, MPS and NSSLs without being crosslinked to SPANb did not accumulate in mouse lung specifically, instead distributed in nude mice without any organ-specificity. Third, pulmonary MPS levels in rats exposed to MPS-NSSLs-SPANbs were 3.81 times of those in rats exposed to conventional MPS, and the AUC_0–12 h_ of MPS in lung tissues of rats exposed to MPS-NSSLs-SPANb was 9.34 times of that of rats exposed to conventional MPS. Furthermore, MPS-NSSLs-SPANbs showed a longer circulation time than MPS in rats, thus possibly reducing the effective dosage. MPS levels in the plasma of rats exposed to MPS-NSSLs-SPANb or MPS-NSSLs were similar to those in the rats exposed to conventional MPS, indicating that crosslink of SPANb to NSSLs may not affect the circulation time of NSSLs (Maruyama et al., 1995). In this study, targeted and non-targeted liposomes can reach the liver and spleen to greater levels than the drug alone. Although toxicity was not observed, there were possible effects on the immune responses in these organs. In the pharmacokinetic part, a small amount of SPA exists in the whole body, mainly in the lung tissue. Targeted liposomes enter the body and reach the liver/spleen with blood circulation. The liver has phagocytosis and immune function, which may intercept the targeted antibody and lead to the increase of drug concentration in the liver. Liposomes in the spleen may be recognized as antigens and activate the immune response. In the future, we might need to pay attention to the impacts of targeting and non-targeting liposomes in the immune responses of these organs.

GC is the main therapeutic drug to treat AE-IPF. However, because GC does not accumulate in the lung specifically and pulmonary GC concentration is low, to achieve satisfactory efficacy for AE-IPF, physicians usually administer high-dose GC to patients with AE-IPF, unavoidably causing GC-associated side effects, including infection, diabetes mellitus, hypertension, osteoporosis, non-healing wound, and Cushing's syndrome. Therefore, some patients with AE-IPF may not die from AE-IPF but from high-dose GC-associated severe side effects, such as infection. MPS-NSSLs-SPANb of the current study, which targeted the lung specifically and effectively, could overcome the limitations of clinical application of GC. The current study found that MPS-NSSLs-SPANb at regular dose (1 mg/kg/d) and low-dose (0.5 mg/kg/d) attenuated pulmonary damage rapidly in rats with AE-IPF, reduced pro-inflammatory cytokine levels in BALF, extended rat survival. The low-dose MPS-NSSLs-SPANb (MPS 0.5 mg/kg)+AE-IPF group in fact had the highest 10-week survival rate. Our findings indicate that MPS-NSSLs-SPANb may reduce the effective therapeutic dosage of MPS, achieve better efficacy, and reduce GC-associated side effects. In addition, MPS-NSSLs-SPANb from the current study did not cause obvious liver and kidney toxicity in rats and did not increase infection, suggesting that MPS-NSSLs-SPANb appear to be safe.

MPS-NSSLs-SPANb were first prepared successfully, and the agent targeted lung tissue specifically and effectively. MPS-NSSLs-SPANb showed better efficacy and low toxicity than conventional MPS in rats with AE-IPF.

## Supplementary Material

Supplemental Material

## Data Availability

The data that support the findings of this study are available on request from the corresponding author. The data are not publicly available due to privacy or ethical restrictions.
