# Supplementary material for: Development and assessment of the efficacy and safety of human lung-targeting liposomal methylprednisolone crosslinked with nanobody
Source: Drug Deliv. 2021 Jul 5;28(1):1419–31. doi: 10.1080/10717544.2021.1921073 (PMC8259875; doi:10.1080/10717544.2021.1921073)
Supplement: Supplemental Material [file IDRD_A_1921073_SM2359.docx]

# Supplementary files

Including supplementary Figures (Figure S1 and S2) and Tables (Table S1-S7).


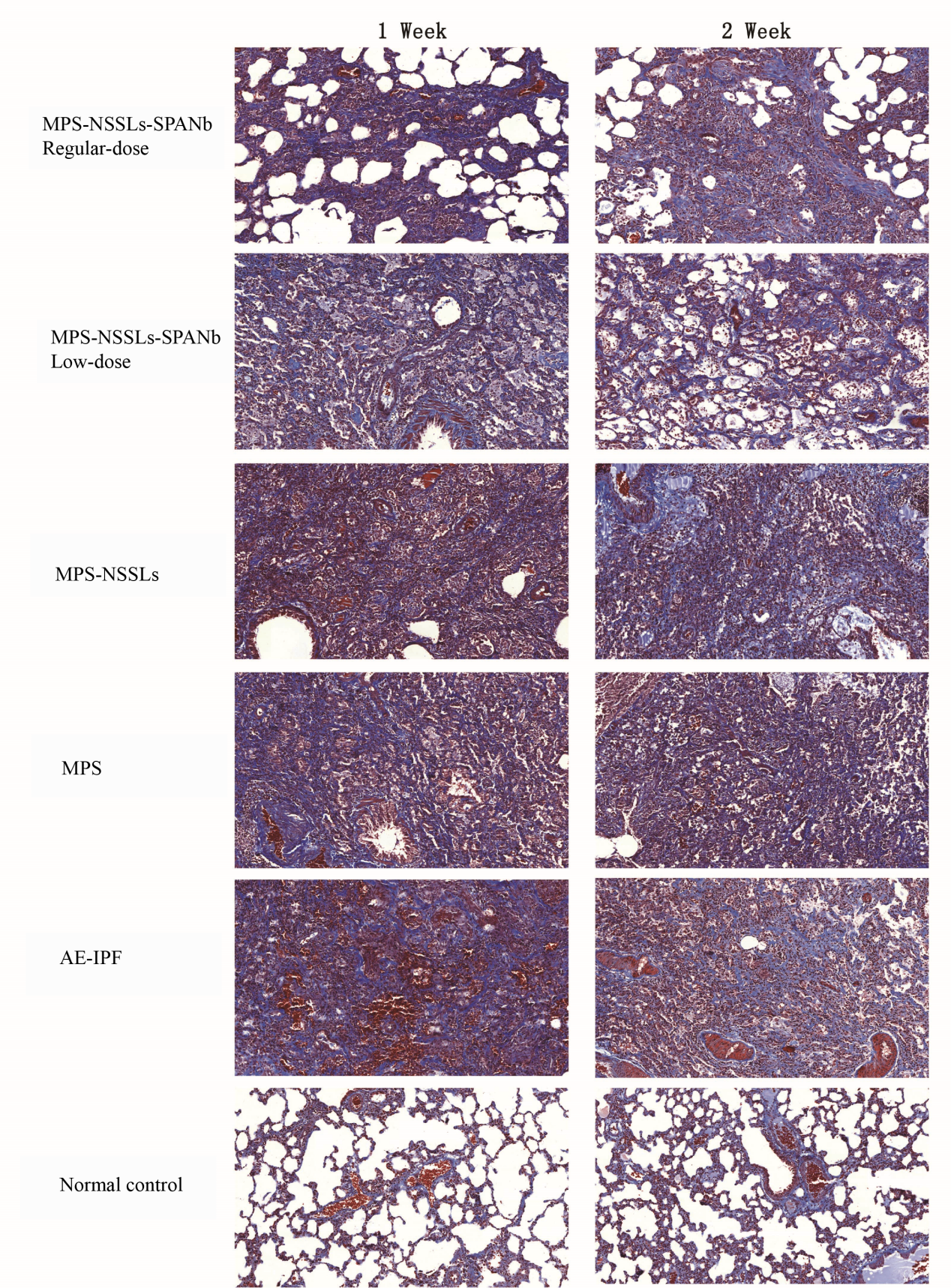


Figure S1 Images of Masson staining of rat lung tissues. The magnification was 20x.


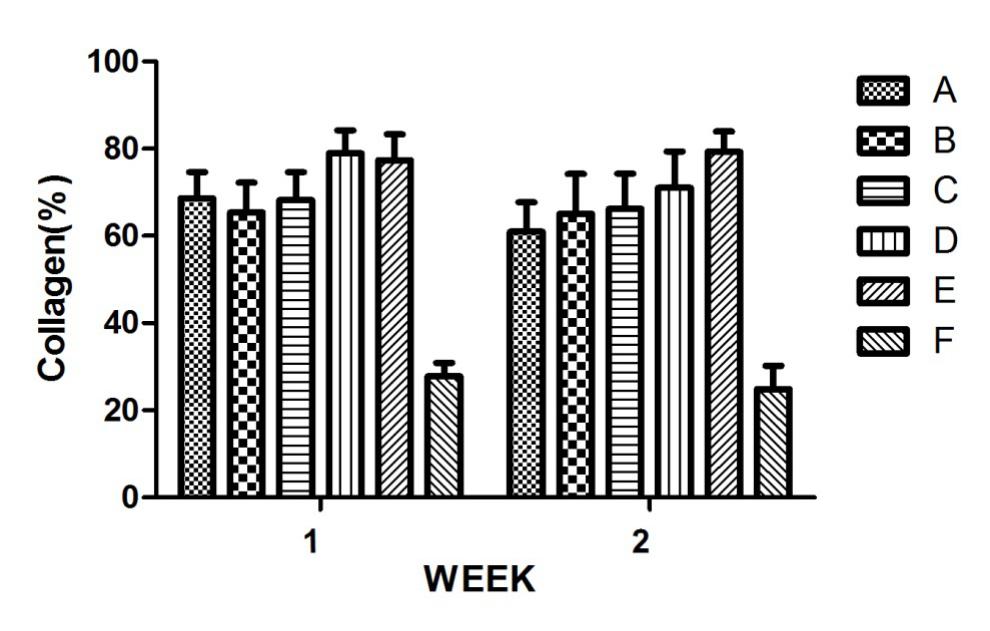


Figure S2 Score of Masson staining. A: Regular-dose MPS-NSSLs-SPANb+AE-IPF group; B: low-dose MPS-NSSLs-SPANb+AE-IPF group; C: MPS-NSSLs+AE-IPF group; D: MPS+AE-IPF group; E: AE-IPF group; F: normal control group.

**Tables**

**Table S1 Encapsulation efficiency of MPS-NSSLs**

| **Time** | **Encapsulation efficiency %*** |
| --- | --- |
| Week 0 | 90.06 ± 0.32 |
| Week 4 | 88.7 ± 1.5 |
| Week 8 | 87.1 ± 1.81 |
| Week 12 | 83.4 ± 3.4 |

* EE%: Encapsulation efficiency %. EE = [(MPS_total_) – (MPS_free_)] ÷ (MPS_total_).

MPS_total_: Total MPS amount. MPS_free_: free MPS.

**Table S2 AUC_0-12h_ and Cmax of MPS in plasma and different tissues**

| **Specimen** | **AUC_0-12h/μg·ml_^-1^_·h_** | | | **C_max_/_μg·ml_^-1^** | | |
| --- | --- | --- | --- | --- | --- | --- |
|  | MPS | MPS-NSSLs | MPS-NSSLs-SPANb | MPS | MPS-NSSLs | MPS-NSSLs-SPANb |
| Plasma | 22.77 | 74.87 | 67.31 | 12.86 | 16.90 | 16.85 |
| Lung | 17.16 | 48.21 | 158.19 | 6.99 | 14.98 | 26.68 |
| Liver | 31.04 | 92.07 | 103.76 | 11.88 | 20.64 | 26.37 |
| Spleen | 17.95 | 56.52 | 62.69 | 7.83 | 17.23 | 21.01 |
| Kidney | 132.34 | 127.84 | 95.66 | 30.72 | 18.65 | 19.96 |
| Heart | 10.04 | 9.45 | 7.89 | 2.40 | 2.62 | 1.89 |

AUC_0-12h_: area under the concentration-time ROC curve in 12 hours.

C_max_: peak concentration of MPS.

**Table S3 The accumulation of MPS-NSSLs and MPS-NSSLs-SPANb in plasma and different tissues**

| Specimen | Re | | Ce | |
| --- | --- | --- | --- | --- |
|  | MPS-NSSLs | MPS-NSSLs-SPANb | MPS-NSSLs | MPS-NSSLs-SPANb |
| Plasma | 3.29 | 2.95 | 1.31 | 1.31 |
| Lung | **2.81.** | **9.22** | **2.14** | **3.81** |
| Liver | 2.97 | 3.31 | 1.73 | 2.22 |
| Spleen | 3.14 | 3.49 | 2.20 | 2.68 |
| Kidney | 0.97 | 0.12 | 0.61 | 0.39 |
| Heart | 0.94 | 0.79 | 1.09 | 0.79 |

Re: relative uptake rate. Re = (AUC)a ÷ (AUC)b. AUC: area under ROC curve. Ce: ratio of peak concentration. Ce = (Cp)a ÷ (Cp)b. Cp represents peak concentration. “a” represents MP-NSSL-SPANb or MP-NSSL group; “b” represents MP group.

**Table S4 Liver and kidney toxicity of MPS-NSSLs-SPANb,** **MPS-NSSLs, and MPS in rats**

| Group | Exposure | ALT (IU/L) | AST (IU/L) | Cr (mmol/L) | BUN (umol/L) |
| --- | --- | --- | --- | --- | --- |
| A: regular-dose MPS-NSSLs-SPANb+AE-IPF | 1 week | 38.13±4.029 | 110.0±22.34 | 20.13±1.016 | 8.371±0.605 |
|  | 2 weeks | 39.50±4.326 | 105.3±8.500 | 16.67±2.604 | 8.257±1.098 |
| B: low-dose MPS-NSSLs-SPANb+AE-IPF | 1 week | 45.29±4.765 | 104.3±20.74 | 22.17±1.139 | 7.417±0.195 |
|  | 2 weeks | 36.14±3.019 | 106.3±13.04 | 19.57±2.937 | 7.743±0.512 |
| C: MPS-NSSLs+AE-IPF | 1 week | 35.00±3.124 | 120.7±18.49 | 26.91±1.389 | 7.529±0.340 |
|  | 2 weeks | 35.14±3.225 | 101.8±8.964 | 16.37±0.785 | 7.373±0.394 |
| D: MPS+AE-IPF | 1 week | 33.33±2.319 | 137.4±34.24 | 23.65±1.025 | 7.757±0.358 |
|  | 2 weeks | **57.00±7.528^*^** | 87.43±5.546 | **29.29±3.355^*^** | 7.420±0.790 |
| E: AE-IPF | 1 week | 33.50±3.284 | 90.40±8.286 | 26.97±1.683 | **9.717±0.705^*^** |
|  | 2 weeks | **51.17±4.262^*^** | 71.80±6.996 | **25.98±2.932^*^** | 7.994±0.653 |
| F: Normal control | 1 week | 28.75±5.391 | 75.00±18.20 | 20.48±3.440 | 6.775±0.948 |
|  | 2 weeks | 33.40±4.501 | 90.00±10.46 | 14.45±1.136 | 6.090±0.977 |

* represents significantly different vs. F: the normal control group, *P* < 0.05. Multiple group comparison was analyzed by ANOVA. *P* < 0.05 was considered significantly different.

**Table S5 Culturing bronchoalveolar lavage fluid to test bacterial and fungal infection**

| Group | One-week exposure | Two-week exposure |
| --- | --- | --- |
| A: regular-dose MPS-NSSLs-SPANb+AE-IPF | no | One positive *S. epidermidis* |
| B: low-dose MPS-NSSLs-SPANb+AE-IPF | no | no |
| C: MPS-NSSLs+AE-IPF | One positive *S. epidermidis* | One positive *S. epidermidis* and two positive *E. Coli* |
| D: MPS+AE-IPF | no | three positive *S. epidermidis* |
| E: AE-IPF | no | no |
| F: normal control | no | no |

*S. epidermidis: Staphylococcus epidermidis*.

**Table S6 Drug exposure of each rat group**

| **Group** | **Drug** | **Dosage (MPS mg/kg body weigh)** | **Frequency (/day)** |
| --- | --- | --- | --- |
| A: regular-dose MPS-NSSLs-SPANb+AE-IPF | MPS-NSSLs-SPANb | 1 | 1 |
| B: low-dose MPS-NSSLs-SPANb+AE-IPF | MPS-NSSLs-SPANb | 0.5 | 1 |
| C: MPS-NSSLs+AE-IPF | MPS-NSSLs | 1 | 1 |
| D: MPS+AE-IPF | MPS | 1 | 1 |
| E: AE-IPF | Saline | 0^*^ | 1 |
| F: normal control | Saline | 0^*^ | 1 |

*represents that same amount of saline was injected via the tail vein.

**Table S7 PCR primer sequences**

| **Species** | **Gene** | **Forward sequence (5′-3′)** | **Reverse sequence (5′-3′)** |
| --- | --- | --- | --- |
| Rat | β-Actin | CACCATTGGCAATGAGCGGTTC | AGGTCTTTGCGGATGTCCACGT |
| Rat | NF-κB | AGAACTTCCCAGGTGGACTG | CTGGTGGCTGGTAATGTCAG |
